# One-step synthesis of a multi-functional imidazole luminescent sensor with AIE, acid-responsive, and sulfate-sensing properties

**DOI:** 10.1039/d6ra01574c

**Published:** 2026-04-21

**Authors:** Pawan Kumar

**Affiliations:** a Department of Chemistry, Govt. Degree College Doda UTJ&K 182202 India pawantandon169@gmail.com

## Abstract

A novel TBM-based chemosensor exhibiting multi-stimuli-responsive photophysical behaviour has been designed and systematically investigated through combined experimental and computational studies. In solution, the sensor displays distinct intramolecular charge-transfer (ICT) absorption and emission, with aggregation-induced emission (AIE) observed in mixed solvents due to restriction of intramolecular motion. Pronounced acidochromic behaviour upon protonation and selective recognition of HSO_4_^−^ result in enhanced and red-shifted absorption. In the solid state, TBM exhibits bright yellow emission and mechanochromic luminescence arising from mechanically induced changes in molecular packing. Density functional theory and time-dependent density functional theory calculations provide insight into the electronic structure, charge-transfer characteristics, and stimulus-induced modulation of the photophysical properties. These findings demonstrate that the TBM scaffold effectively integrates ICT, aggregation effects, and external-stimuli sensitivity, highlighting its potential for chemical sensing and stimuli-responsive luminescent applications.

## Abstract 2

A novel TBM-based chemosensor exhibiting multi-stimuli-responsive photophysical behaviour has been designed and systematically investigated through combined experimental and computational studies.

## Graphical abstract

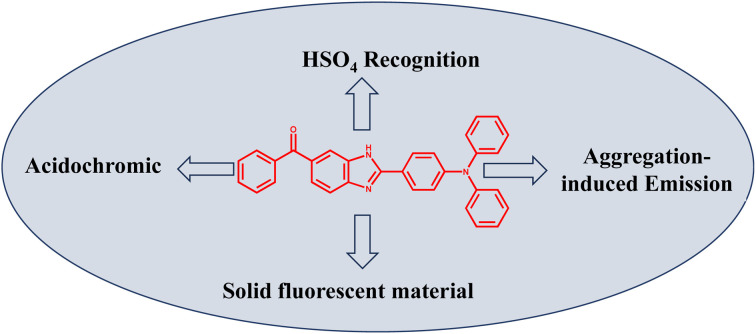

## 1 Introduction

The development of multifunctional fluorescent chemosensors has attracted significant attention due to their potential applications in chemical sensing, bioimaging, and optoelectronic devices.^1–5^ Fluorescent sensors that respond to multiple stimuli-such as solvent polarity, pH, ions, and mechanical forces-offer versatile platforms for real-time monitoring and smart material design. Among various sensing mechanisms, intramolecular charge transfer (ICT) and aggregation-induced emission (AIE) are particularly effective in producing large Stokes shifts and enhanced emission efficiency in both solution and solid states.^6–10^

Hydrogen sulfate (HSO_4_^−^) is a biologically and environmentally important anion, and its selective detection remains challenging due to interference from other anions and the need for high sensitivity.^11,12^ Protonation-sensitive acidochromic sensors also provide complementary optical responses, enabling the study of microenvironmental changes and proton-mediated interactions.^13–15^ Additionally, mechanochromic luminescence in the solid state offers a convenient visual readout for mechanical stimuli, expanding the utility of fluorescent materials in optoelectronic and stress-sensing applications.^16^

Computational studies have emerged as an indispensable tool for elucidating the mechanisms underlying such multi-stimuli-responsive behaviour. Density functional theory (DFT) and time-dependent DFT (TD-DFT) can predict electronic structures, frontier orbital distributions, and excited-state properties, providing insight into ICT processes, aggregation effects, and stimulus-induced modulation of photophysical properties. By combining experimental measurements with computational analysis, it is possible to rationally design sensors with enhanced selectivity, tunable emission, and multi-modal responsiveness.^17^

In the present study, we report a TBM-based chemosensor that integrates multiple stimulus-responsive behaviours within a single molecular scaffold. The sensor exhibits ICT-driven absorption and emission, aggregation-induced emission in mixed solvents, pronounced acidochromism upon protonation, selective HSO_4_^−^ recognition, and mechanochromic luminescence in the solid state. In parallel, computational studies using density functional theory (DFT) and time-dependent DFT (TD-DFT) have become indispensable for understanding the electronic structure, excited-state properties, and stimulus-dependent photophysical behaviour of such systems. These results demonstrate the versatility of the TBM scaffold for multi-stimuli-responsive luminescent applications.

## 2 Experimental

### 2.1. Materials and procedures

All solvents employed in this work-ethyl acetate (EA), acetonitrile (CH_3_CN), dimethyl sulfoxide (DMSO), methanol (MeOH), ethanol (EtOH), tetrahydrofuran (THF), dichloromethane (DCM), and toluene (TOL)-were of spectroscopic grade and purchased from Spectrochem India. Triphenylamine carboxaldehyde and 3,4-diaminobenzophenone were obtained from Sigma-Aldrich and used as received, without further purification. Stock solutions of the synthesized chromophores were prepared in DCM, methanol, or acetonitrile at a concentration of 1.0 mM and subsequently diluted to micromolar levels as required for spectroscopic and other experimental investigations.

### 2.2. Instrumentation

Fourier-transform infrared (FTIR) spectra were acquired using an Agilent Technologies Cary 630 FTIR spectrometer over the range of 400–4000 cm^−1^ at 25 °C. Nuclear magnetic resonance (NMR) spectra were recorded on Bruker BioSpin AVANCE III HD (500 MHz) and JEOL-FT NMR AL (400 MHz) instruments, using tetramethylsilane (TMS) as the internal reference and CDCl_3_ as the deuterated solvent. Chemical shift values (*δ*) are reported in parts per million (ppm) relative to residual solvent peaks. High-resolution mass spectra (HRMS) of the chromophores were obtained using a Bruker micrOTOF II LC-MS system. UV-visible absorption spectra were measured with a Shimadzu UV-1800 spectrophotometer using a 1 cm path-length quartz cuvette. Fluorescence emission spectra were recorded on Horiba Fluorolog and PerkinElmer LS-55 spectrofluorometers.

The dynamic light scattering (DLS) studies were carried out on a MALVERN Zetasizer Nano ZS instrument. Photoluminescence quantum yields (PLQYs) in the solid state were determined using an integrating sphere attached to a spectrofluorometer. Samples were measured under ambient conditions, and absolute quantum yields were calculated from the ratio of emitted to absorbed photons with appropriate correction for background and instrumental response (eqn (S1) in SI).

### 2.3. Computational specification

All theoretical calculations were performed using the Gaussian 09 software package. The ground-state geometries of the chromophores were fully optimized using density functional theory (DFT) with the hybrid B3LYP functional. The 6-31G(d,p) basis set was applied to all constituent atoms (C, H, O, and N). Excited-state properties were investigated using time-dependent density functional theory (TD-DFT), and the lowest 30 singlet excited states were calculated.^18,19^ Molecular orbitals and electron density distributions were visualized using Gauss View 5.0.9.

### 2.4. Experimental section

#### 2.4.1. Synthesis of triphenylamine-1*H*-benzoimidazole(phenyl)methanone (TBM)

Triphenylamine carboxaldehyde (0.50 g, 1.0 mmol) and 2,3-diaminobenzophenone (0.38 g, 1.0 mmol) were dissolved in ethanol (3 mL). To this mixture, three drops of acetic acid were added as a catalyst, and the reaction was stirred at room temperature for 3 h. The progress of the reaction was monitored by thin-layer chromatography (TLC) using hexane/ethyl acetate (7 : 3, v/v) as the eluent. Upon completion, the resulting precipitate was collected by filtration and dried under reduced pressure using a rotary evaporator to afford TBM as a solid in 91% yield. ^1^H NMR (500 MHz, CDCl_3_, 25 °C) *δ*/ppm: 7.13 (m, 8H, 8× ArH), 7.28 (t, *J* = 7.5, 4H, 4× ArH), 7.43 (t, *J* = 7.5, 2H, 2× ArH), 7.54 (m, 2H, 2× ArH), 7.80 (m, 3H, 3× ArH), 7.96 (d, *J* = 8.5, 2H, 2× ArH), 8.09 (s, 1H, 1× ArH). ^13^C NMR (125 MHz, CDCl_3_, 25 °C) *δ*/ppm: 121.67, 124.14, 125.48, 127.91, 128.18, 129.54, 130.01, 132.02, 138.35, 146.83, 150.15. HRMS: *m*/*z* calculated for C_32_H_23_N_3_O: 466.1875. Found: 466.1977.

## 3 Results and discussion

### 3.1. Synthesis

**Scheme 1:**
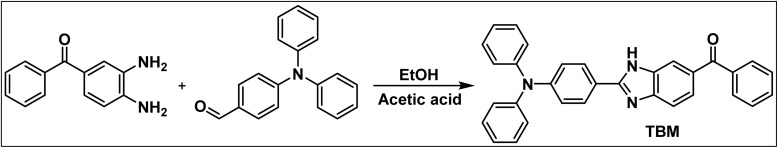
Synthesis of imidazole based chemosensor TBM.

The imidazole-based chemosensor TBM was synthesized *via* a condensation–cyclization reaction between triphenyl carboxaldehyde and 2,3-diaminobenzophenone. The structure of TBM was fully characterized using standard spectroscopic techniques, including ^1^H NMR, ^13^C NMR, and mass spectrometry. All spectral data are consistent with the proposed molecular structure and are provided in the Experimental section and SI (see Fig. S1–S3 and Scheme 1).

### 3.2. Absorption and emission characteristics in dichloromethane

**Fig. 1:**
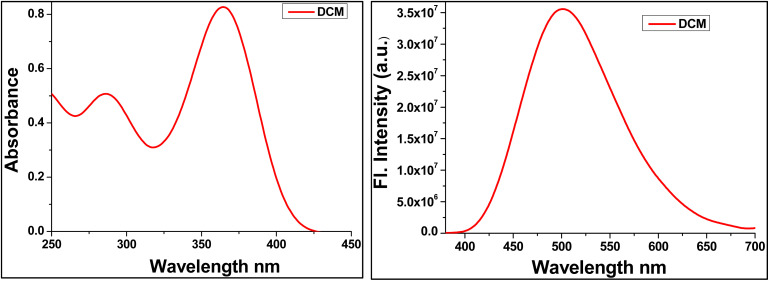
UV-visible absorption and emission spectra of TBM (1 × 10^−5^ M) in dichloromethane.

The UV-vis absorption spectrum of the TBM chemosensor recorded in dichloromethane (DCM) exhibits two well-defined absorption bands centered at 287 and 365 nm (Fig. 1), indicative of multiple electronic transitions within the molecular framework. The higher-energy band at 287 nm is assigned to a localized n–π* transition, primarily involving the π-conjugated or aromatic segments of the molecule. This transition represents intrafragment electron density redistribution with minimal charge-transfer character. In contrast, the lower-energy absorption at 365 nm corresponds to an intramolecular charge-transfer (ICT) transition or π–π*, arising from electronic communication between the electron-rich donor and electron-deficient acceptor moieties. The red-shifted position of this band relative to the π–π* transition suggests enhanced conjugation and partial charge separation in the excited state, consistent with donor–acceptor systems.^20^

**Fig. 2:**
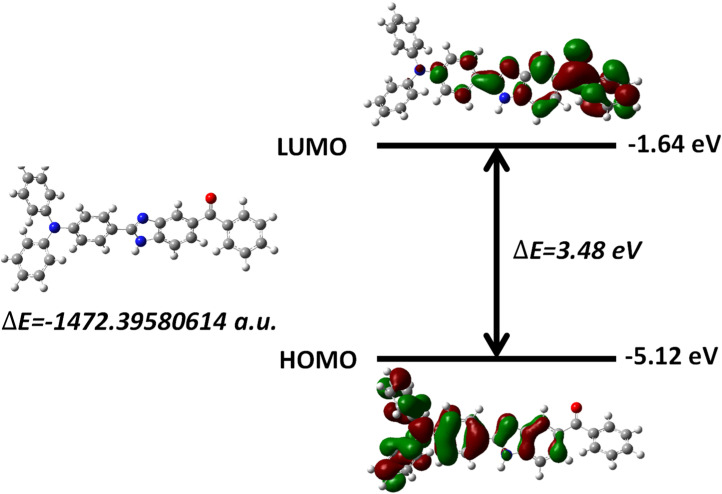
TD-DFT electron density of HOMO–LUMO of TBM depicting ICT.

Density functional theory (DFT) calculations support these experimental assignments. The computed frontier molecular orbitals reveal that the HOMO is localized over the donor and π-conjugated regions, while the LUMO is predominantly distributed over the acceptor core. This spatial arrangement results in a HOMO → LUMO transition with significant intramolecular charge transfer (ICT) character, in excellent agreement with the experimentally observed absorption at 365 nm (Fig. 2). The calculated HOMO–LUMO energy gap of 3.48 eV closely matches the experimental value of 3.39 eV, further validating the computational model. The clear separation of the frontier orbitals confirms that excitation induces electron density transfer from the donor to the acceptor, stabilizing the excited state.^21^

**Fig. 3:**
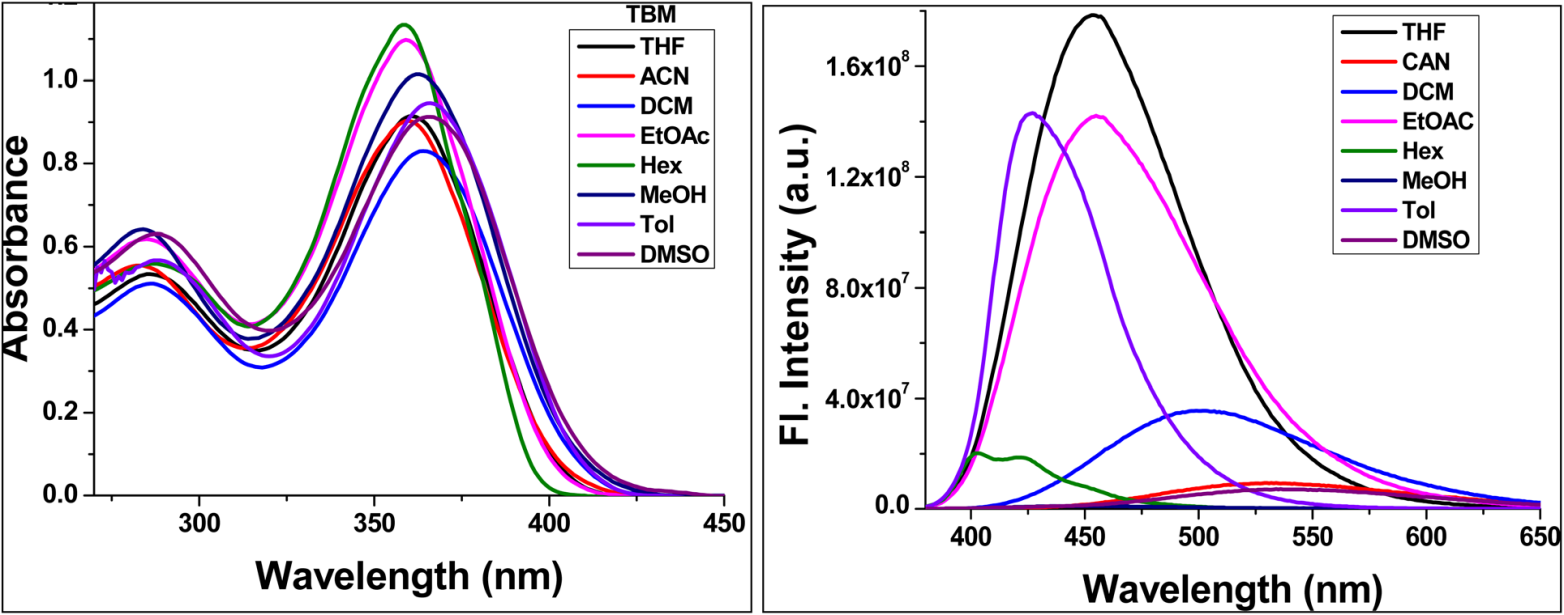
UV-visible and fluorescence spectra of TBM were recorded in various polarity of solvents at 10 µM concentration. Excitation wavelength; *λ*_ex_ = 360 nm.

Upon photoexcitation, the chemosensor exhibits a broad emission band centered at 520 nm in DCM, corresponding to a large Stokes shift (Fig. 3). Such a pronounced shift reflects substantial excited-state relaxation prior to photon emission. Excitation into either the π–π* or ICT absorption bands is followed by rapid internal conversion, funneling the population to the lowest-energy singlet excited state, which is predominantly ICT in nature. Time-dependent DFT (TD-DFT) calculations indicate that this relaxed excited state undergoes geometrical reorganization, leading to increased planarity and enhanced charge separation between donor and acceptor segments (Fig. 1). The structural relaxation stabilizes the excited state, lowering its energy and producing the observed red-shifted emission. Additionally, the polar aprotic environment of DCM contributes to stabilization of the ICT state through dipolar solvation, without strong hydrogen-bonding interactions. This balance facilitates efficient radiative decay while preserving significant charge-transfer character.

The TBM chemosensor also demonstrates pronounced solvatochromic behavior, with emission maxima shifting from 403 nm in hexane to 538 nm in DMSO, depending on solvent polarity (Fig. 3). This behavior can be rationalized through modulation of ICT upon photoexcitation. In nonpolar solvents, the electronic distribution remains largely localized, resulting in higher-energy emission. In contrast, polar solvents stabilize the excited-state dipole *via* solvation, promoting ICT and inducing a bathochromic shift. UV irradiation further accentuates bathochromic shifts in the absorption spectrum, reflecting enhanced π–π* and n–π* transitions facilitated by the polarizable environment. TD-DFT calculations corroborate this interpretation, showing significant spatial redistribution of the frontier molecular orbitals in the excited state. Calculated excitation energies correlate well with experimental trends, confirming that solvent polarity modulates the HOMO–LUMO gap and governs the optical response.

Overall, the combined absorption, emission, and solvatochromic studies indicate that TBM possesses dual electronic character, with localized π–π* transitions and ICT processes shaping its photophysical properties. The large Stokes shift, efficient ICT emission, and pronounced solvent-dependent behavior highlight its potential as a polarity-responsive chemosensor, with TD-DFT providing mechanistic insight at the molecular level.

### 3.3. Aggregation-induced emission (AIE) behavior

**Fig. 4:**
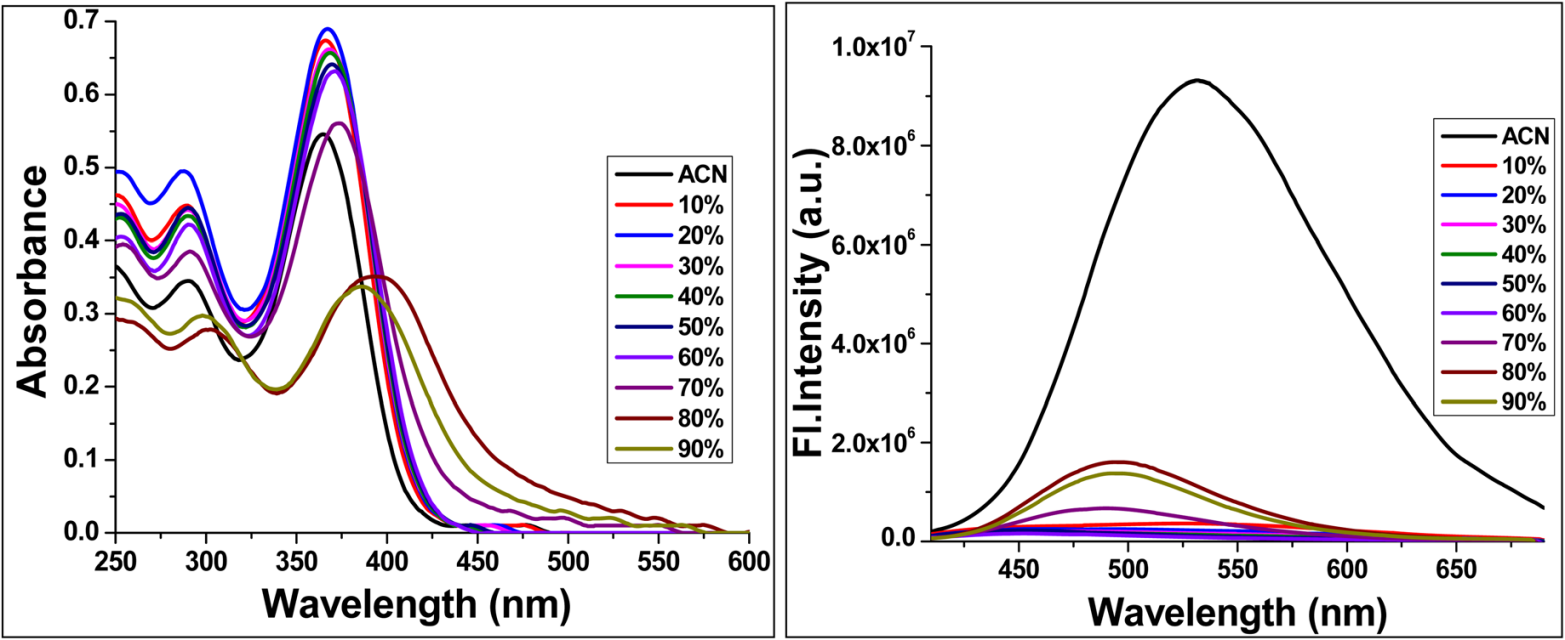
Water fraction studies of TBM from 0–90% (water fraction in acetonitrile).

The aggregation-induced emission (AIE) characteristics of TBM were investigated in acetonitrile–water mixtures to assess the effect of aggregation on its photophysical properties (Fig. 4). In pure acetonitrile, a good solvent, TBM exists predominantly as monomeric species, exhibiting an absorption maximum at 365 nm and an emission maximum at 532 nm. In this solvated state, intramolecular rotations and vibrations provide non-radiative decay pathways, resulting in moderate fluorescence intensity.

As the water fraction is increased to 90%, the solubility of TBM decreases, leading to aggregate formation. This is reflected in a red shift of the absorption to 395 nm and a blue-shifted yet significantly enhanced emission at 496 nm. The enhanced fluorescence arises from restriction of intramolecular motions within the aggregates, which suppresses non-radiative decay. Additionally, aggregation stabilizes the intramolecular charge-transfer (ICT) excited state, further modulating the emission energy.^22–24^

These observations demonstrate that TBM exhibits classic aggregation-induced emission (AIE) behaviour, displaying weak emission in good solvents due to freely rotating molecular units, while showing significantly enhanced fluorescence in poor solvents as a result of aggregate formation. This behaviour can be attributed to the restriction of intramolecular motion (RIM) in the aggregated state, along with stabilisation of the intramolecular charge transfer (ICT) state, which collectively govern the observed solvent-dependent spectral shifts and emission enhancement. Such characteristics highlight the potential of TBM as a stimuli-responsive luminescent material.

To further substantiate the aggregation process, dynamic light scattering (DLS) measurements were carried out for TBM at a concentration of 1 × 10^−5^ M in CH_3_CN/H_2_O mixtures with varying water fractions (Fig. S4 in SI). In a 40 : 60 (v/v) CH_3_CN : H_2_O mixture, nanoaggregates with an average hydrodynamic diameter of 273 nm were observed, along with a moderate polydispersity index (PDI) of 0.090. Increasing the water fraction to 80% (20 : 80, v/v) resulted in a marked decrease in aggregate size to 184 nm, accompanied by a narrower size distribution (PDI = 0.175). A similar average size of 182 nm was obtained at 90% water content (10 : 90, v/v), although with a slightly broader distribution (PDI = 0.092). The reduction in aggregate size with increasing water fraction suggests enhanced aggregation in aqueous-rich environments, driven by decreased solubility, which is consistent with the observed AIE characteristics.

### 3.4. Viscosity-dependent emission behavior

**Fig. 5:**
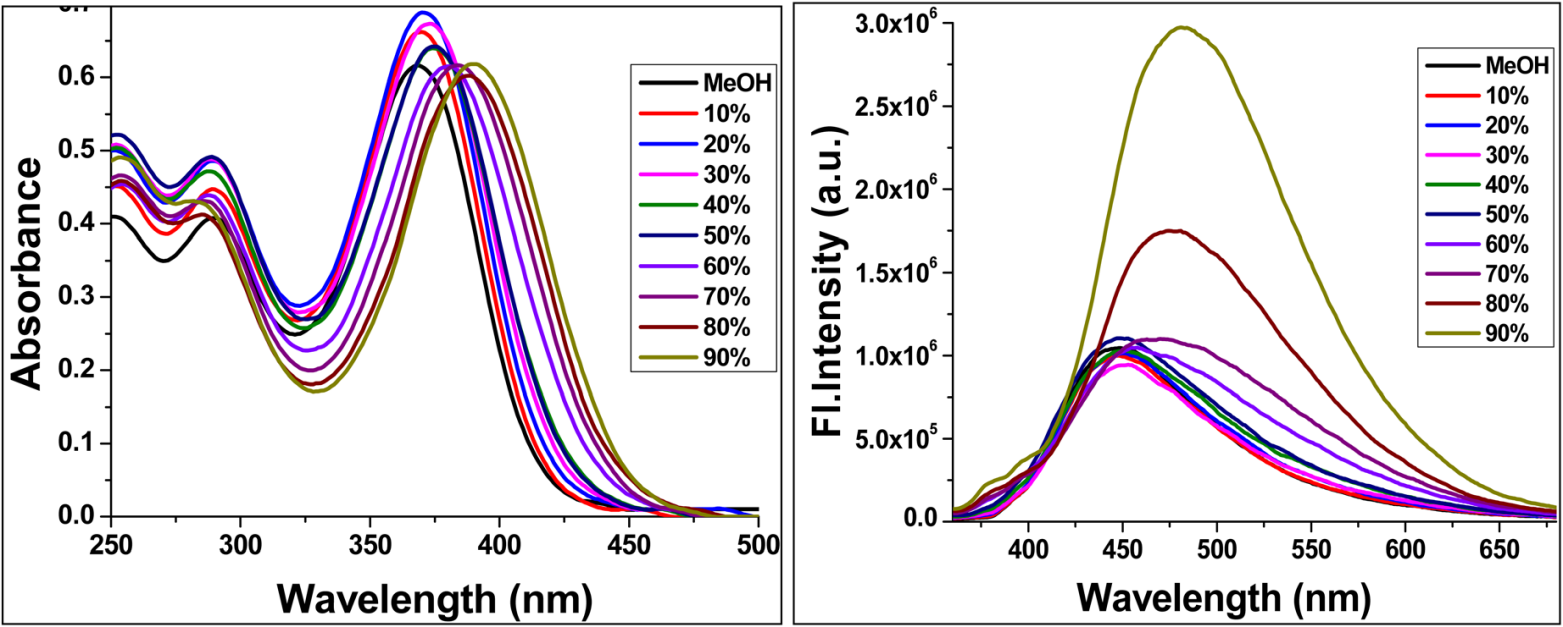
Absorbance and fluorescence emission spectrum chemosensor TBM in MeOH and glycerol at 1 × 10^−5^ M.

The photophysical response of TBM is strongly influenced by solution viscosity, which was probed using glycerol–methanol mixtures (Fig. 5). As the glycerol content increases, the viscosity of the medium rises, leading to a progressive red shift in the absorption maximum from 368 to 390 nm under UV studies. This shift reflects changes in the local solvent environment and the stabilization of the excited state due to restricted molecular motions in a more viscous medium.

Similarly, fluorescence measurements show that the emission maximum shifts from 448 to 481 nm with increasing glycerol fraction. The enhanced viscosity restricts intramolecular rotations and vibrations, which are common non-radiative decay pathways in less viscous solvents. By limiting these molecular motions, the excited-state energy is preserved, leading to more efficient radiative decay and an increase in emission intensity.^25–27^

Overall, these observations demonstrate that the fluorescence of TBM is highly sensitive to the viscosity of its surrounding environment. Notably, TBM exhibits a fluorescence quantum yield (*Φ*_F_) of 0.08 in methanol (0.544 cP), which increases significantly to 0.44 in a glycerol/methanol mixture (80% v/v, 250 cP). This pronounced enhancement indicates that restricted molecular motion in more viscous media effectively suppresses non-radiative decay pathways, thereby promoting radiative emission. The increase in viscosity hinders intramolecular rotations and vibrations, leading to stabilization of the ICT excited state and enhanced fluorescence intensity. Collectively, these findings highlight the strong viscosity-dependent emission behavior of TBM, underscoring its potential as a sensitive probe for micro-viscosity and constrained molecular environments.

### 3.5. Anion sensing behavior of TBM in acetonitrile

**Fig. 6:**
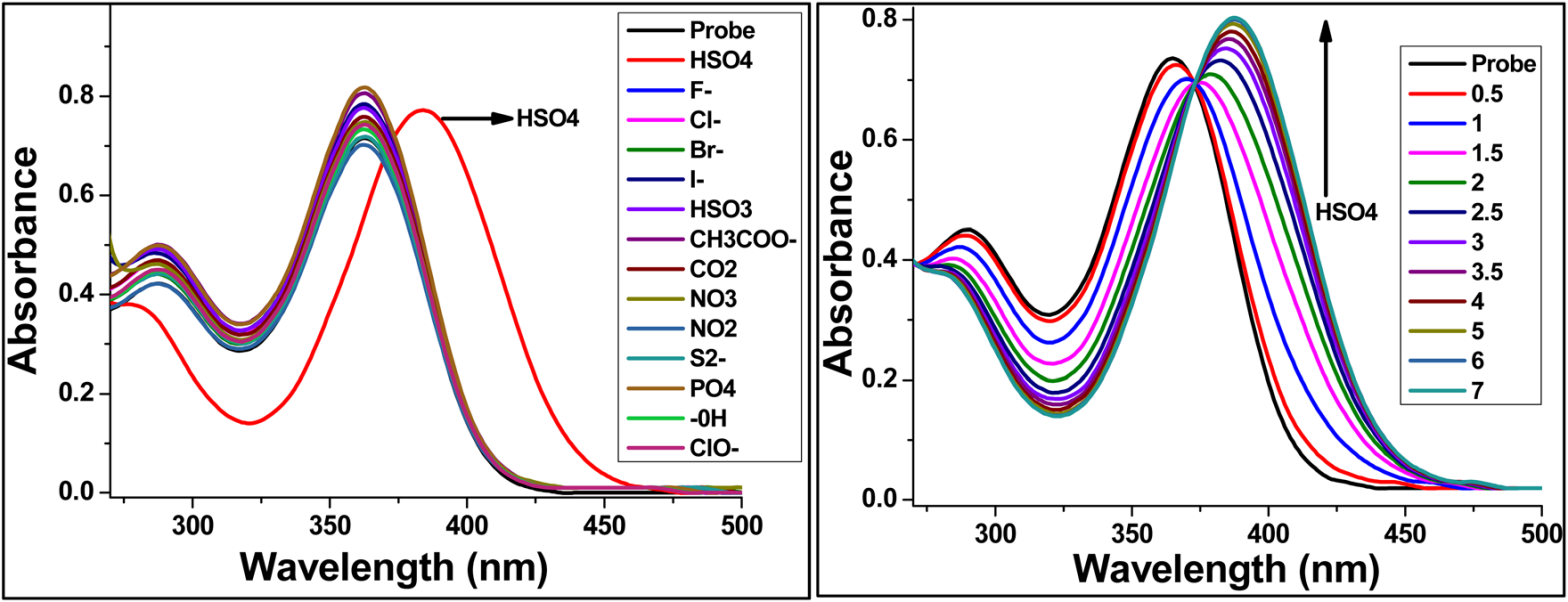
UV-visible titration spectrum of chemosensor TBM at (1 × 10^−5^ M) in DCM with addition of anion and hydrogen bisulfate.

The anion-sensing capability of TBM was evaluated in acetonitrile, focusing on its response toward various anions (Fig. 6). Upon titration with hydrogen sulfate (HSO_4_^−^), the UV-vis absorption spectrum exhibited a bathochromic shift of the main absorption band from 365 to 384 nm (Fig. 6). This red shift became pronounced upon the addition of 7 equivalents of HSO_4_^−^, indicating a strong and selective interaction between TBM and the hydrogen sulfate anion.

No comparable spectral changes were observed upon the addition of other common anions, demonstrating the high selectivity of TBM toward HSO_4_^−^. The observed bathochromic shift suggests that binding of the anion perturbs the electronic environment of the chemosensor, likely through hydrogen bonding or electrostatic interactions, which stabilizes the intramolecular charge-transfer (ICT) excited state. This stabilization lowers the energy of the π–π* transition, leading to the observed red shift in absorption.^28,29^

These results highlight the ability of TBM to act as a selective optical probe for HSO_4_^−^ in acetonitrile, where the spectral response is clearly distinguished from other anions. The magnitude of the bathochromic shift and its correlation with anion concentration demonstrate that the interaction is both strong and stoichiometrically well-defined, making TBM a promising candidate for anion recognition applications in organic solvents.

### 3.6. Solid-state emission and mechanochromic behavior

**Fig. 7:**
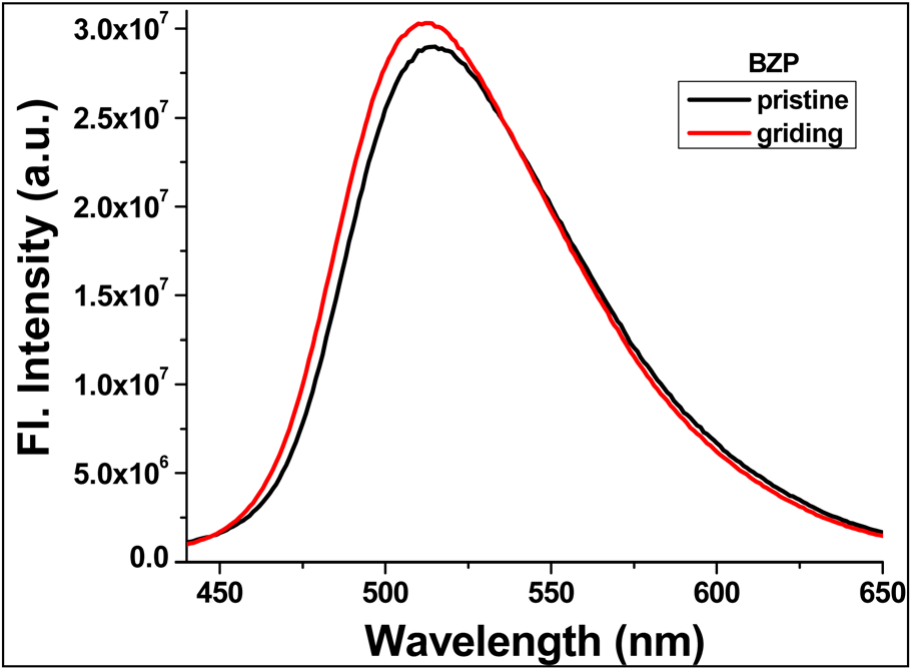
Solid-state emission spectrum of chemosensor TBM recorded at room temperature.

The TBM chemosensor exhibits strong light-yellow fluorescence in the solid state, with an emission maximum at 519 nm, indicating efficient radiative decay in the condensed phase (Fig. 7). Solid-state measurements further reveal a fluorescence quantum yield of up to 0.34, confirming its bright emission characteristics. This pronounced solid-state emission is attributed to the sterically bulky and non-planar molecular structure, which limits close π–π stacking and suppresses aggregation-induced quenching. In crystalline or semi-crystalline arrangements, weak non-covalent interactions and hydrogen bonding, create a rigid molecular packing that restricts intramolecular motions, stabilizing the excited state and promoting efficient fluorescence. Accordingly, the observed emission is assigned to a solid-state intramolecular charge-transfer (ICT) excited state stabilized by the constrained environment.

Upon mechanical grinding, TBM displays mechanochromic luminescence, evidenced by a slight blue shift of the emission maximum to approximately 510 nm. This response arises from the disruption of the ordered molecular packing and partial conversion to an amorphous phase, which alters intermolecular interactions and modifies excited-state relaxation pathways. The retention of emission in a similar spectral region indicates that the molecular emissive unit remains intact, confirming that the observed mechanochromism is due to packing reorganization rather than chemical degradation.^30,31^

These observations underscore the importance of mechanically tunable intermolecular interactions in dictating the solid-state photophysical properties of TBM. The combination of robust solid-state fluorescence and reversible mechanochromic behavior highlights the potential of TBM as a stimuli-responsive luminescent material for optoelectronic and sensing applications.

### 3.7. Acidochromic behavior of TBM

**Fig. 8:**
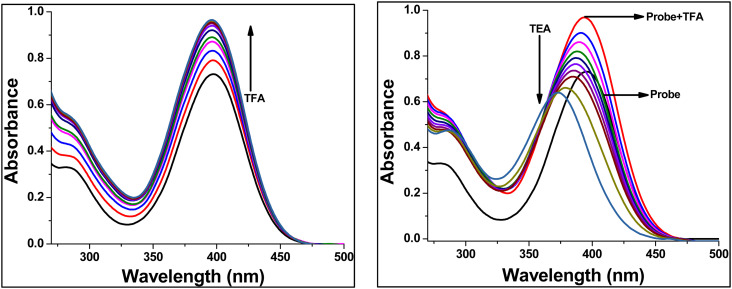
UV-visible titration spectrum of chemosensor TBM at (1 × 10^−5^ M) in DCM with subsequential addition of TFA and TEA.

The proton-responsive photophysical behavior of TBM was investigated in dichloromethane (DCM) by monitoring changes in the UV-vis absorption spectrum upon incremental addition of trifluoroacetic acid (TFA) and subsequent neutralization with triethylamine (TEA) (Fig. 8). Addition of TFA led to a significant increase in absorption intensity, indicative of protonation of the chemosensor. Protonation occurs predominantly at the imine nitrogen atom of the imidazole ring, which modifies the electronic distribution and enhances the oscillator strength of the π–π* and intramolecular charge-transfer (ICT) transitions, resulting in the observed intensity enhancement.^32^

Subsequent addition of TEA caused a hypsochromic shift of the absorption band from 396 to 373 nm, corresponding to deprotonation of the imine nitrogen. Deprotonation restores the original electronic configuration and partially reduces conjugation within the molecular framework, giving rise to the observed blue shift. This process is fully reversible, demonstrating that TBM can switch controllably between protonated and neutral states.

These results confirm that TBM functions as a reversible acid-base-responsive optical sensor, where protonation stabilizes the ICT state and enhances absorption, while deprotonation reverses this effect. The distinct spectral changes associated with protonation and deprotonation highlight the potential of TBM for pH sensing and stimuli-responsive optoelectronic applications.

## 4 Conclusion

A TBM-based chemosensor exhibiting multi-responsive photophysical behaviour has been developed and systematically studied. The sensor displays intramolecular charge-transfer characteristics, aggregation-induced emission, acidochromism, and selective HSO_4_^−^ recognition, resulting in pronounced modulation of its absorption and emission properties. In the solid state, bright yellow fluorescence and mechanochromic luminescence are observed due to mechanically induced changes in molecular packing. Overall, the versatile optical response of the TBM scaffold highlights its potential for applications in chemical sensing and stimuli-responsive luminescent materials.

## Conflicts of interest

The authors declare no conflicts of interest.

## Supplementary Material

RA-016-D6RA01574C-s001

## Data Availability

All data supporting the findings of this study are available within the article and its supplementary information (SI). Additional data related to this work are available from the corresponding author upon reasonable request. Supplementary information: spectral data (^1^H and ^13^C NMR, and HRMS), size distribution measured by the dynamic light scattering (DLS) spectrum and quantum yield calculations method. See DOI: https://doi.org/10.1039/d6ra01574c.
