# Supplementary material for: One-step synthesis of a multi-functional imidazole luminescent sensor with AIE, acid-responsive, and sulfate-sensing properties
Source: RSC Adv. 2026 Apr 21;16(22):20489–97. doi: 10.1039/d6ra01574c (PMC13098485; doi:10.1039/d6ra01574c)
Supplement: RA-016-D6RA01574C-s001 [file RA-016-D6RA01574C-s001.pdf]

Supplementary Information (SI) for RSC Advances.
This journal is © The Royal Society of Chemistry 2026

One-Step Synthesis of a Multi-Functional Imidazole Luminescent Sensor with AIE, Acid-Responsive, and Sulfate-Sensing Properties

Pawan Kumar*

Department of Chemistry, Govt. Degree College, Doda, UTJ&K, 182202 India.
E-mail: pawantandon169@gmail.com

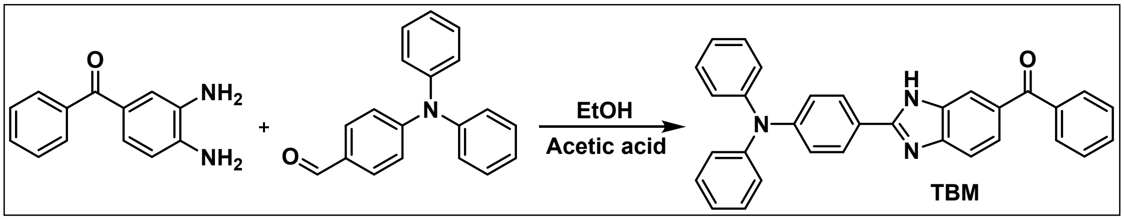

Scheme :1. Synthesis of imidazole based chemosensor TBM.

1. Experimental section

Synthesis of triphenylamine-1*H*-benzoimidazole(phenyl)methanone (TBM).

The mixing triphenylamine carboxaldehyde (0.5 g. 0.001 mol) and 2,3-diaminobenzophenone (0.38 g, 0.001 mol) in 3 ml ethanol is accomplished by adding three drops of acetic acid as a catalyst and stirring at room temperature for three hours. Completion of reaction confirmed through TLC in hexane: ethyl acetate (7:3% v/v), as well as filtering and drying the precipitate over the rotavapor. Product **TBM** was obtained in 91% yield. ^{1}H NMR (500 MHz, $CDCl_3$, 25 ^{0}C) δ/ppm: 7.13 (m 8H, 8 × ArH), 7.28 (t, *J* =7.5, 4H, 4 × ArH),) 7.43 (t, *J* =7.5, 2H, 2 × ArH) 7.54 (m 2H, 2 × ArH),7.80 (m 3H, 3 × ArH) 7.96 (d, *J* =8.5, 2H, 2 × ArH) 8.09 (s, 1H, 1 × ArH). ^{13}C NMR (125 MHz, $CDCl_3$, 25 ^{0}C) δ/ppm: 121.67, 124.14, 125.48, 127.91, 128.18, 129.54, 130.01, 132.02, 138.35, 146.83, 150.15. HRMS: *m/z* calculated for $C_{32}H_{23}N_3O$: 466.1875. Found: 466.1977.

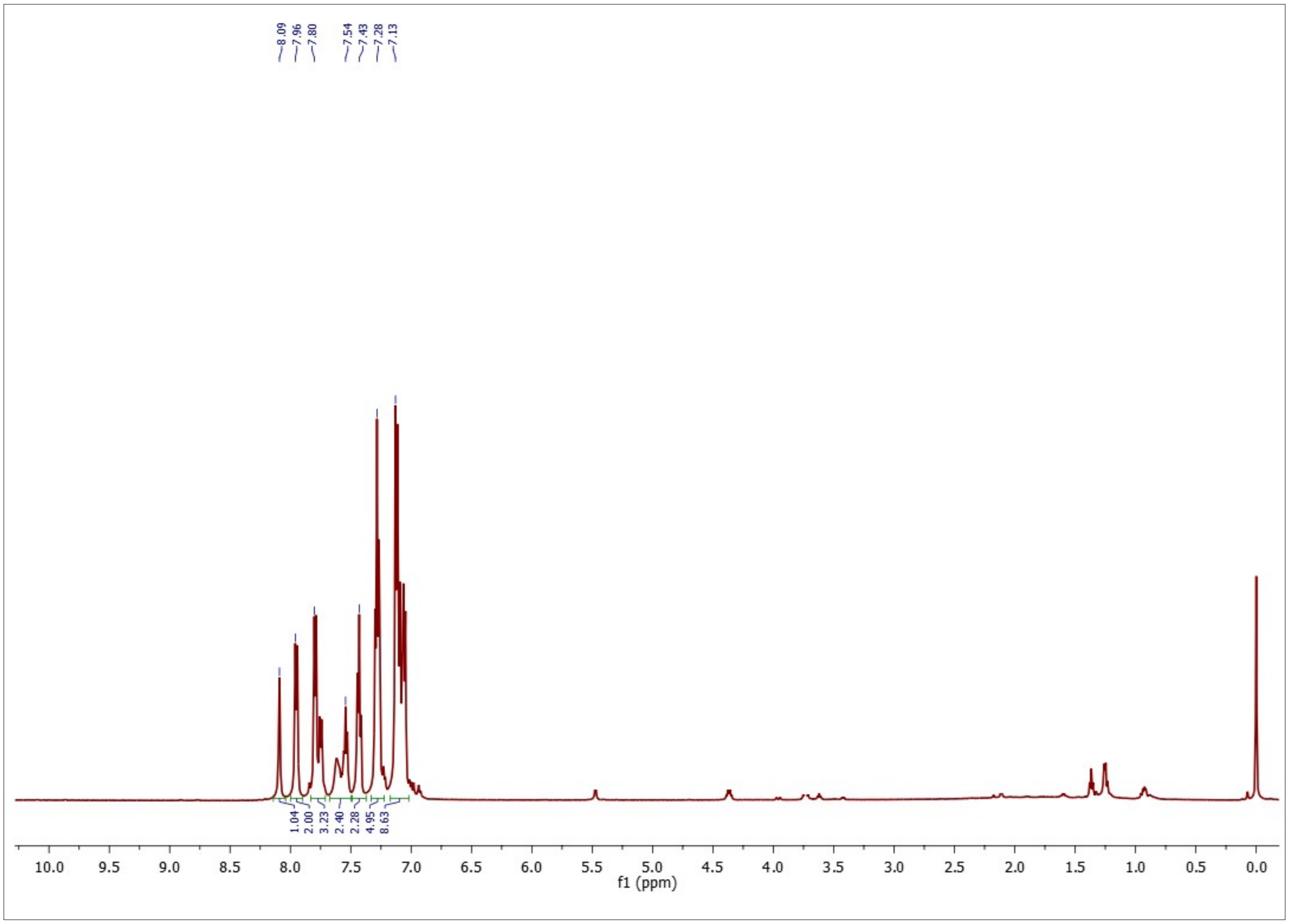

Figure S1. ^{1}H NMR (500 MHz, $CDCl_3$) of **TBM**.

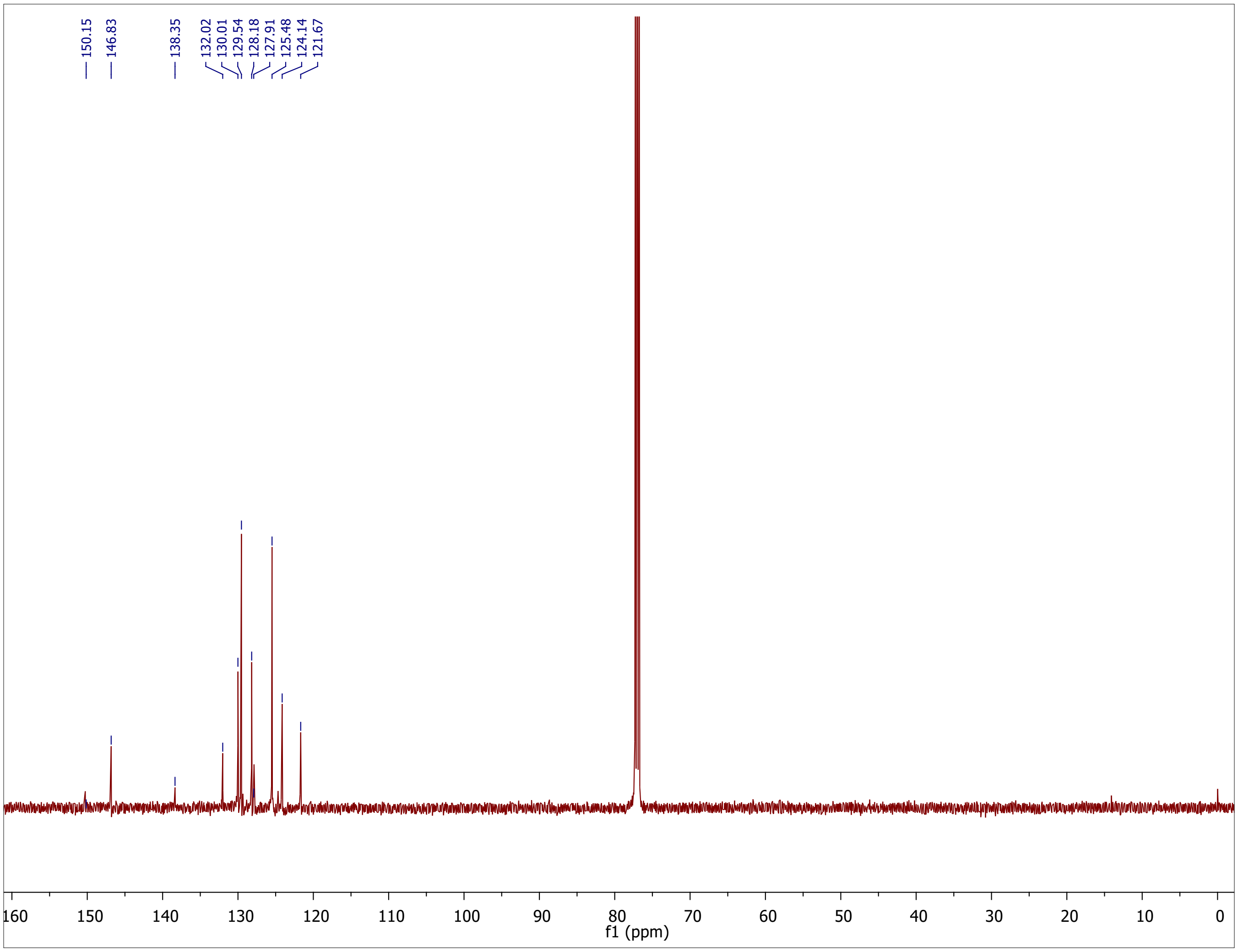

Figure S2. ^{13}C NMR (500 MHz, $CDCl_3$) of **TBM.**

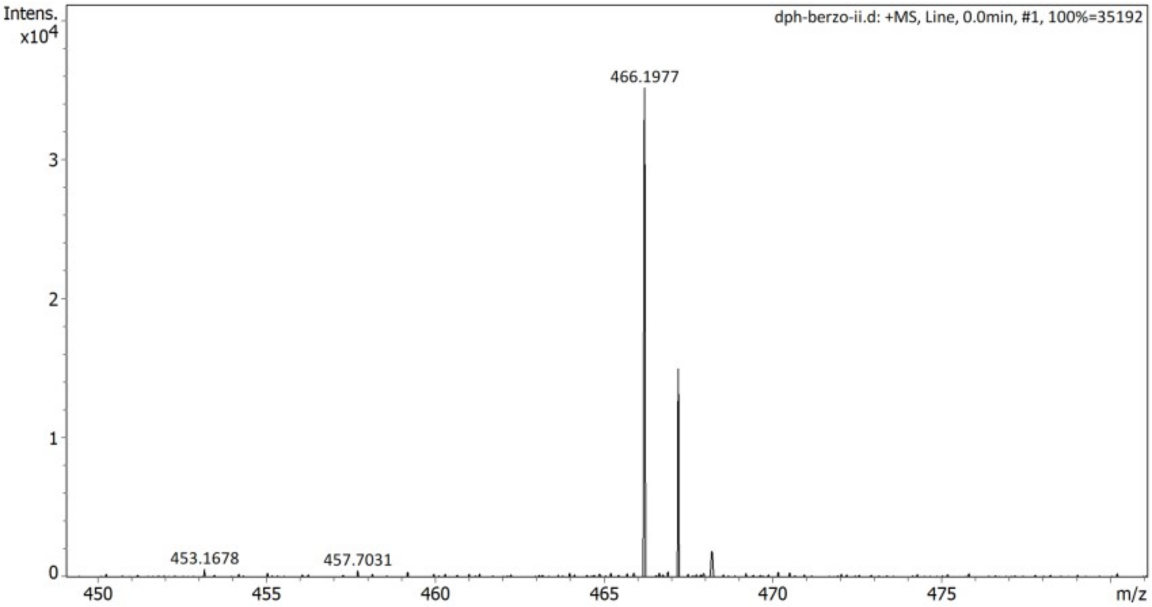

Figure S3. HRMS spectrum of **TBM.**

Dynamic Light Scattering (DLS) Method

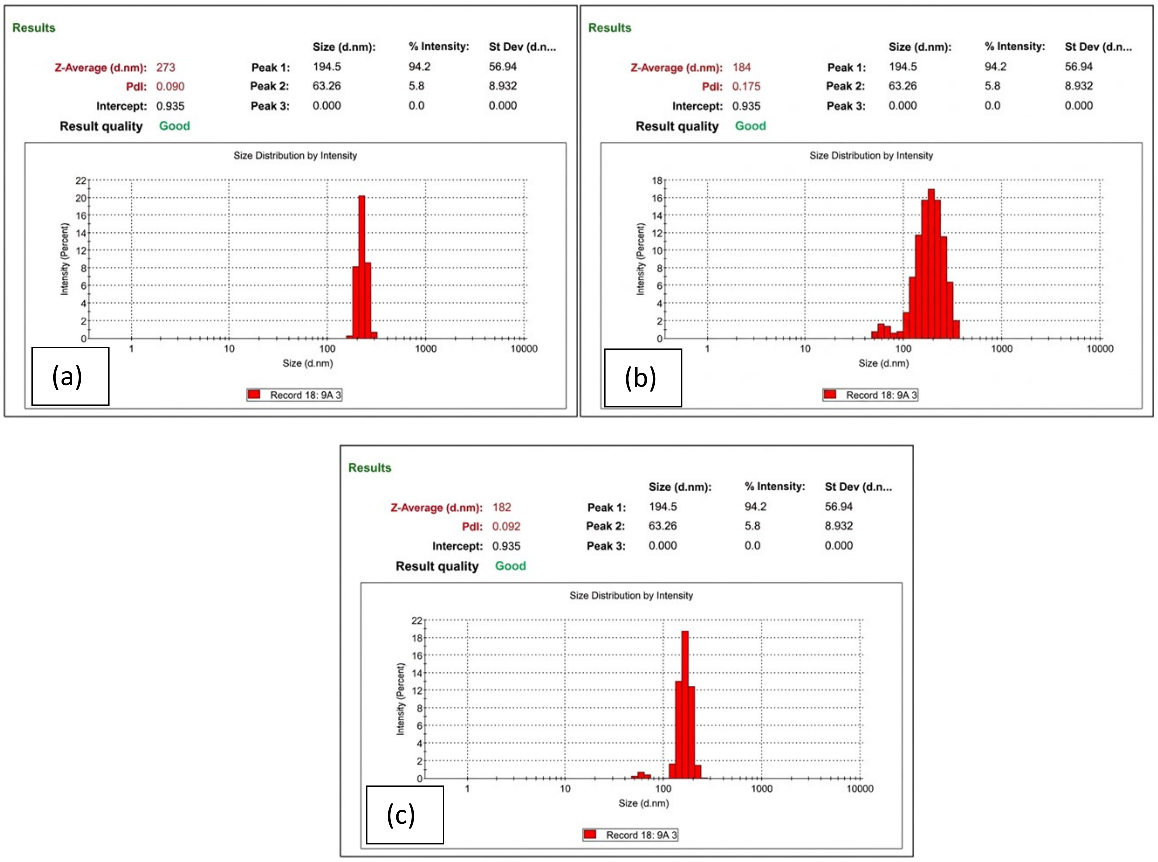

Figure S4. Size distribution measured by the dynamic light scattering (DLS) method for TBM (1 × 10^{-5} M) in different fractions of water in CH_3CN: (a) CH_3CN:H_2O = 40:60 (v/v), (b) CH_3CN:H_2O = 20:80 (v/v), and (c) CH_3CN:H_2O = 10:90 (v/v). The average particle sizes were 273, 184, and 182 nm, with corresponding polydispersity index (PDI) values of 0.090, 0.175, and 0.092, respectively.

Quantum yield calculations

The fluorescence quantum yields were calculated using equation 1. Quinine sulphate having quantum yield of 0.54 in 0.1M of sulphuric acid was used as standard.

$$\phi_S = \frac{1 - 10^{-A_{ref}} \times I_S \times \eta_S^2}{1 - 10^{-A_S} \times I_{ref} \times \eta_{ref}^2} \times \phi_{ref} \qquad (S1)$$

I_S and I_{ref} represent the area under the fluorescence spectral curve of the sample and the reference, respectively. A_S and A_{ref} are optical densities of the sample and the reference compounds, respectively at the excitation wavelengths and η is the refractive index of the solvents.
